# Does the type of first-line regimens influence the receipt of second-line chemotherapy treatment? An analysis of 3211 metastatic colon cancer patients

**DOI:** 10.1002/cam4.176

**Published:** 2014-01-07

**Authors:** Zhiyuan Zheng, Nader Hanna, Eberechukwu Onukwugha, Emily S Reese, Brian Seal, C Daniel Mullins

**Affiliations:** 1 Department of Pharmaceutical Health Services Research, University of Maryland School of PharmacyBaltimore, MD; 2 Department of Surgery, Division of General and Oncologic Surgery, University of Maryland School of MedicineBaltimore, MD; 3 Bayer Healthcare Pharmaceuticals Inc.Wayne, NJ

**Keywords:** Chemotherapy/biologics treatment, inverse probability weighting Cox regression, metastatic colon cancer, receipt of second-line treatment, SEER-Medicare, treatment history

## Abstract

With new agents entering the market, the sequencing of first-line (Tx1), second-line (Tx2), and subsequent chemotherapy/biologics regimens are being examined. We examined how Tx1 regimens impacted the likelihood of receiving Tx2 among metastatic colon cancer (mCC) patients. Surveillance, Epidemiology and End Results (SEER)-Medicare data were used to identify elderly mCC patients between 2003 and 2007. The inverse probability weighting Cox regression method was utilized to study the relationship between receipt of Tx2 and Tx1 regimens, controlling for patient-level factors. Of the 7895 elderly patients identified, 3211 (41%) received Tx1 of which 1440 proceeded to Tx2. The impact of Tx1 on receipt of Tx2 varied by the specific regimens utilized. As compared to 5FU/LV users, IROX (Hazard Ratio [HR] = 0.03; *P* < 0.01) and IROX + Biologics (HR = 0.20; *P* < 0.01) users were less likely to receive Tx2; (oxaliplatin) OX + Biologics (HR = 1.26; *P* < 0.01) users were more likely to receive Tx2. Significant patient-level factors included: Hispanic ethnicity (HR = 0.67; *P* < 0.01); being married (HR = 0.87; *P* = 0.01); proxy for poor performance status (HR = 0.82; *P* = 0.05); each 10-year age increment (HR = 1.14; *P* < 0.01); and State buy-in status (HR = 1.21; *P* = 0.01). The specific first-line regimen does impact mCC patients' likelihood of receiving Tx2 in clinical practice. Elderly mCC patients, their health care providers, and policy makers will benefit from new evidence about the impact of sequencing of treatment lines.

## Introduction

Most metastatic colon cancer (mCC) patients should receive chemotherapy with/out biologics as the primary treatment. Only a small number of mCC patients who have limited liver or lung metastasis are suitable for curative surgical resection. While mCC is incurable in most cases, chemotherapy aids with palliating symptoms, prolonging progression-free and overall survival, and improving patients' quality of life. Chemotherapy may consist of first-line treatment (Tx1), second-line treatment (Tx2), and subsequent line treatment (TxS). It has been demonstrated that Tx2 improved overall survival as well as time to progression among mCC patients 1–10. Moreover, the addition of biologics in Tx2 was associated with prolonged overall survival and progression free survival 11–13. A systematic literature review of the effectiveness and safety of chemotherapy in elderly colon cancer patients suggests that the effectiveness of treatment is no different than in younger populations, after controlling for performance status 14. The recommended management strategies are based on evidence from randomized clinical trials (RCTs). However, which subgroup of elderly mCC patients are getting Tx2 and why some patients do not proceed to Tx2 are not clearly described in the literature and not addressed by available treatment guidelines.

The National Comprehensive Cancer Network (NCCN) colon cancer guideline identifies a list of recommended chemotherapy drugs and biologics 15. Fluorouracil, capecitabine (pro-drug of fluorouracil), floxuridine (pro-drug of fluorouracil), leucovorin, and levoleucovorin were grouped together and referred as 5FU/LV, which became the standard of care for mCC patients in the 1990s. 5FU/LV showed improvement in overall survival, as compared to the best alternative supportive care for mCC patients 16,17. Another two chemotherapy drugs, that is irinotecan (IRI) and oxaliplatin (OX), can be administered to mCC patients either alone or in addition to 5FU/LV. Both IRI and OX were proven to improve response rates, progression-free survival and overall survival, as compared to 5FU/LV alone 18–22. IRI and OX, referred to as IROX, can also be administered together and given to mCC patients. The use of biologics, that is bevacizumab (BEV), cetuximab (CETUX), and panitumumab (PANIT), in addition to chemotherapy drugs showed further improvement in progression-free survival and overall survival 23.

The choice of regimens in Tx1 may directly affect a patient's likelihood of proceeding to Tx2 subsequently. However, there is little empirical evidence that examines how initial treatment affects the likelihood of receiving Tx2 among elderly mCC patients. Moreover, patient-level factors may also impact the utilization of Tx2. Therefore, a better understanding of the various dynamics between Tx1 regimens, patient-level factors and receipt of Tx2 can help clinicians talk with patients regarding the available treatment options. For example, a clinician may spend more time with unmarried patients if they are less likely to either receive more aggressive regimens in Tx1 or proceed to Tx2 as compared to married patients. Also, the same patient-level factor (e.g., comorbid conditions, race/ethnicity, age, and socioeconomic status, etc.) may impact Tx2 differently than Tx1. This is because the reasons for proceeding to Tx2 might be different than the reasons for initiation of Tx1 24.

## Methods

### Patients, dataset, and inclusion/exclusion criteria

This study used the Surveillance Epidemiology and End Results (SEER)-Medicare data to identify elderly patients diagnosed with mCC between 2003 and 2007. This study sample consisted of patients (age 66 and above at the time of diagnosis) with complete Medicare coverage, that is Medicare Part A and B. A full year of claims data prior to cancer diagnoses was required for the accurate ascertainment of baseline comorbidities; therefore, those who were enrolled in a managed care plan within 1 year prior to cancer diagnosis were excluded. Patients diagnosed post mortem were also excluded. The remaining patients were followed up through death or the end of 2009. Subjects were censored from the study upon loss of complete Medicare coverage, end of study, or enrollment in a managed care plan.

### Identification of first- and second-line treatment

The algorithm used for identifying treatment lines was developed by Bikov et al. 25. It is a claims-based algorithm for mCC patients that uses SEER-Medicare data to identify treatment line based on the treatment(s) received and the timing of treatments.

The NCCN recommended chemotherapy drugs for mCC patients during the study period were 5FU/LV, IRI and OX; the recommended biologics were BEV, CETUX, and PANIT. In our analysis, we created a categorical variable to assign mCC patients into different groups according to the regimen utilized in the initial treatment. Patients who had 5FU/LV alone in Tx1 were the reference group. This is because 5FU/LV is historically the standard of care for mCC with a relatively low level of toxicity, as compared to other drugs. Other groups: (1) IRI without biologic(s); (2) OX without biologic(s); (3) IROX without biologic(s); (4) 5FU/LV with biologic(s); (5) IRI with biologic(s); (6) OX with biologic(s); (7) IROX with biologic(s); and (8) biologic(s) alone. Patients were excluded from the analysis if they only had non-NCCN recommended regimens in Tx1 (i.e., if they were not on one of the above regimens). This grouping strategy has clear layers with regard to the toxicity types and levels: 5FU/LV is generally considered to be the least toxic drug among all chemotherapy drugs; IRI and OX are more toxic as compared to 5FU/LV, which limits their use as Tx1 among elderly patients with poor performance status.

### Patient-level factors

Age was defined as a continuous variable (each 10-year increment in age at diagnosis). Race/ethnicity was categorized into Non-Hispanic white, African American, Hispanic, Asian and others. State buy-in status was defined as whether a patient had any State buy-in coverage within 1 year prior to diagnosis, and is indicative of low socioeconomic status. Household median income was defined in $10,000s and it measured the income level of the neighborhood defined by the zip code of the patient's residence. It is an additional proxy for patients' socioeconomic status as has been done in prior studies 26. Charlson comorbidity index (CCI) was created using claims within 1 year prior to cancer diagnosis. An indicator was created to detect any hospital bed use, oxygen use, walking aid use or wheelchair use within 3 months prior to cancer diagnosis, and it was referred as the proxy for poor performance status. Contextual variables, that is year of diagnosis (2003–2007) and the SEER registry sites, were also included in the analysis.

### Statistical analysis

#### Three-category and nine-category grouping strategies

To examine the distributions of Tx1 regimens by select patient-level factors, we first regrouped the detailed nine-category into three broad categories: (1) 5FU/LV; (2) IRI, OX, or IROX; and (3) with biologics. The broad three-category grouping strategy is an intermediate step, which summarizes the detailed nine-category grouping strategy and avoids statistical analyses of samples with small cell sizes. When we included the no treatment group, there were four mutually exclusive groups of patients in comparison.

#### Inverse probability weighting Cox regression

The inverse probability weighting (IPW) Cox regression framework was used to study the receipt of Tx2. Patients intrinsically had different mortality risks at the time of diagnosis and may delay or remain on Tx1 for a long time period, resulting in different opportunities to receive Tx2. The IPW method can be used to adjust each patient's survival probability within the Cox regression framework 27,28. In this study, it gave individual patients different weights according to their probabilities of living longer than the median time from diagnosis to Tx2; therefore, the biases of the regression estimates introduced by patients' mortality risks were reduced by the IPW Cox regression framework. Moreover, some patients were censored in this study (i.e., drop out of study, end of study, and switching to a managed care plan). These censored patients could receive Tx2 elsewhere. Ignoring these patients might introduce bias. Therefore, the probabilities of being censored were also estimated. The final form of IPW consisted of patients' survival probabilities as well as their censoring probabilities.

The association between the receipt of Tx1 and the same set of patient-level factors was also investigated. Therefore, we can compare the impacts of patient-level factors on Tx1 versus Tx2. The statistical significance level was set a priori at *α *= 0.05. The approved Institutional Review Board protocol number is HP-00049426.

## Results

### The identified sample

**Figure 1:**
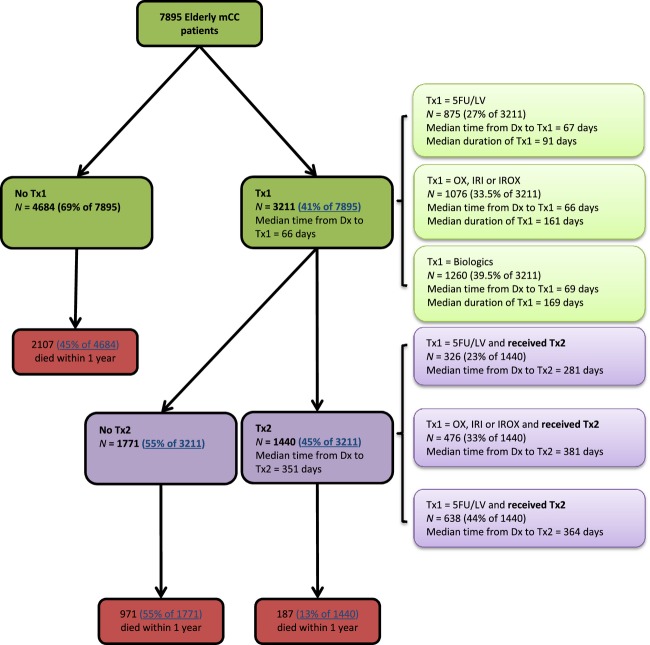
A diagram of elderly metastatic colon cancer (mCC) patients switching to second-line treatment (Tx2).

In our analysis, we identified 7951 elderly mCC patients. One patient was excluded due to high age (above 105). Another 55 patients were excluded due to the use of non-NCCN recommended regimens in Tx1, and 7895 patients remained in the sample. In Figure 1, 3266 (41% of 7895) proceeded to Tx1. The median time from diagnosis to the initiation of Tx1 was about 2 months. Among treated, 1440 (44% of 3266) received Tx2. The median time from diagnosis to the initiation of Tx2 was about 12 months. One-year unadjusted mortality risks were: 13% (187 out of 1440) for patients who proceeded to Tx2; 55% (971 of 1771) for those who had Tx1 only; 45% (2107 of 4684) among untreated patients.

### Distribution of Tx1 drugs and timing of initiations of Tx1 and Tx2

Table 1 shows the distributions of Tx1 regimen(s) by patient-level factors. Among treated, the overall number of patients was (1) 875 (27% of 3211) for the 5FU/LV group; (2) 1076 (34% of 3211) for the OX, IRI and IROX group; (3) and 1260 (39% of 3211) for the biologics group. Figure 1 also shows the time from diagnosis to Tx1 and Tx2, duration of Tx1, respectively. The median time from diagnosis to Tx1 across the three groups was about the same (66–69 days). However, the median duration of Tx1 and the median time from diagnosis to Tx2 were the smallest among the 5FU/LV group (91 and 281 days, respectively) as compared to the OX, IRI or IROX group (161 days and 381 days, respectively) and the biologics group (169 days and 364 days, respectively).

**Table 1:** Distribution of Tx1 chemotherapy drugs/biologics by patient-level factors.

	No treatment			5FU/LV		OX, IRI or IROX			Biologics^2^		
	*N*	Col%	*P*^1^	*N*	Col%	*N*	Col%	*P*^1^	*N*	Col%	*P*^1^
Overall	4684	100	–	875	100	1076	100		1260	100	–
Charlson comorbidity index (CCI)											
CCI = 0	2667	56.9	<.01	533	60.9	717	66.6	<.01	848	67.3	<.01
CCI = 1	1023	21.8		208	23.8	245	22.8		270	21.4	
CCI = 2	530	11.3		82	9.4	71	6.6		91	7.2	
CCI = 3 or above	464	9.9		52	5.9	43	4.0		51	4.1	
Race/ethnicity											
Non-Hispanic White	3665	78.3	<.01	706	80.7	872	81.1	0.02	1037	82.3	0.02
African American	592	12.6		90	10.3	94	8.7		116	9.2	
Hispanic	218	4.7		48	5.5	56	5.2		51	4.1	
Asian	209	4.5		31	3.5	54	5.0		56	4.4	
Proxy for poor performance status											
Yes	601	12.8	<.01	60	6.7	37	3.4	<.01	44	3.5	<.01
No	4083	87.2		815	93.1	1039	96.6		1216	96.5	
Age at diagnosis (mean, SD)	81	7.5	<.01	77	6.4	74	5.3	<.01	74	5.9	0.02
Female vs. male											
Female	2671	57.0	<.01	478	54.7	523	48.6	<.01	637	50.6	<.01
Male	2013	43.0		397	45.3	553	51.4		623	49.4	
Marital status											
Yes	1739	37.1	<.01	449	48.7	679	63.1	<.01	739	58.7	<.01
No	2945	62.9		426	51.3	397	36.9		521	41.3	
Urban living area											
Yes	4196	89.6	<.01	772	88.2	981	91.2	<.01	1129	89.6	0.12
No	488	10.4		103	11.8	95	8.8		131	10.4	
Socioeconomic status											
State buy-in status											
Yes	976	20.8	<.01	126	14.4	113	10.5	<.01	139	11.0	<.01
No	3708	79.2		749	85.6	963	89.5		1121	89.0	
Household median income in $10,000s (mean, SD)	4.8	2.3	0.05	5.1	2.4	5.2	2.5	0.02	5.1	2.3	.98

IRI, irinotecan; OX, oxaliplatin.

1 *P* measures the statistical significance of the difference between the column percentages of the 5FU/LV group and another group (e.g., Biologics vs. 5FU/LV).

2 The biologics group consisted many different combinations of chemotherapy drugs and biologics, and the majority groups are: OX + Biologics (*N* = 792), 5FU/LV + Biologics (*N* = 215), and IRI + Biologics (*N* = 176), which constitute 94% of total 1260 patients.

### Association between age and treatment lines

**Figure 2:**
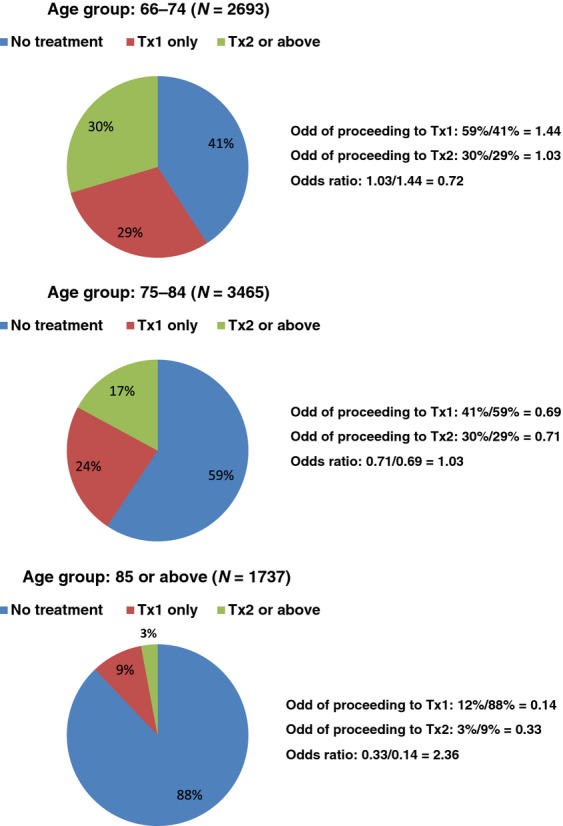
Distribution of treatment lines by age groups.

Figure 2 shows that being older at time of diagnosis is associated with lower likelihoods of receiving any treatment and further treatment, which are represented by the odds of proceeding to Tx1 and Tx2. However, the odds ratio of proceeding to Tx2 versus Tx1 increases from younger to older patients: 0.72 for age group 66–74; 1.03 for age group 75–84; and 2.36 for age group 85 or above. This is likely due to the fact that once an elderly patient is deemed fit to receive Tx1, s/he is likely to proceed to Tx2.

### IPW COX regression results for the receipt of Tx2

Table 2 shows the impact of patient-level factors on receipt of Tx1/Tx2 and how Tx1 regimen(s) affected patients' likelihood of proceeding to Tx2. For Tx2, the regimen(s) received in Tx1 had significant impact on a patient's likelihood of proceeding to Tx2 (as compared to 5FU/LV): (1) IRI and OX were not statistically different than 5FU/LV; (2) IROX was significantly associated with much lower likelihood of proceeding to Tx2 (Hazard Ratio [HR] = 0.03; *P* < 0.01); 3) among the rest, biologic(s) alone (HR = 1.72; *P* = 0.01), and OX + Biologic(s) (HR = 1.26; *P* < 0.01) were associated with higher likelihood of proceeding to Tx2; and IROX + Biologic(s) (HR = 0.20; *P* < 0.01) was associated with lower likelihood of proceeding to Tx2.

**Table 2:** Multivariate correlations among Tx1, patient-level factors, and receipt of Tx2.

	IPW^1^ Cox for Tx1			IPW^1^ Cox for Tx2		
	HR	95% CI	*P*xs	HR	95% CI	*P*
Chemotherapy drugs/biologic(s) in Tx1						
5FU/LV		–		Reference		
IRI		–		0.84	(0.69, 1.01)	0.07
OX		–		0.94	(0.81, 1.08)	0.35
IROX		–		0.03	(0.01, 0.17)	<.01
5FU/LV + Biologic(s)		–		1.05	(0.86, 1.27)	0.65
IRI + Biologic(s)		–		0.89	(0.71, 1.12)	0.33
OX + Biologic(s)		–		1.26	(1.09, 1.46)	<.01
IROX + Biologic(s)		–		0.20	(0.08, 0.53)	<.01
Biologic(s) alone		–		1.72	(1.12, 2.63)	0.01
Charlson comorbidity index (CCI)						
CCI = 0		Reference			Reference	
CCI = 1	1.02	(0.95, 1.10)	0.54	0.97	(0.87, 1.09)	0.60
CCI = 2	0.87	(0.77, 0.99)	0.03	0.88	(0.74, 1.04)	0.13
CCI = 3 or above	0.88	(0.74, 1.04)	0.12	0.87	(0.71, 1.07)	0.20
Proxy for poor performance status	0.61	(0.51, 0.73)	<.01	0.82	(0.68, 1.00)	0.05
Each additional day between Dx and Tx1		–		0.996	(0.995, 0.997)	<.01
Each 10-year increment in age at Dx	0.58	(0.55, 0.61)	<.01	1.14	(1.06, 1.23)	0.01
Female	1.10	(1.04, 1.17)	<.01	1.03	(0.93, 1.13)	0.60
Marital status	1.46	(1.36, 1.56)	<.01	0.87	(0.78, 0.97)	0.01
Urban living area	1.05	(0.94, 1.18)	0.37	0.86	(0.72, 1.02)	0.08
Race/ethnicity						
Non-Hispanic White		Reference			Reference	
African American	0.79	(0.70, 0.88)	<.01	1.13	(0.96, 1.33)	0.15
Hispanic	0.94	(0.82, 1.08)	0.42	0.67	(0.53, 0.84)	<.01
Asian and others	0.93	(0.79, 1.08)	0.33	1.11	(0.88, 1.39)	0.39
Socioeconomic status						
State buy-in status	0.69	(0.62, 0.77)	<.01	1.21	(1.04, 1.40)	0.01
Household median income	1.02	(1.01, 1.04)	<.01	1.00	(0.98, 1.03)	0.72

All patients: *N* = 7895; patients who proceeded to Tx1: *N* = 3211; patients who proceeded to Tx2: *N* = 1440. IPW, inverse probability weighting; Tx1, first-line treatment; Tx2, second-line treatment.

1 The inverse probability weighting method adjusts each mCC patient's probability of living long enough to receive Tx1, Tx2 and TxS.

As for other patient-level factors, higher CCI was generally associated with lower likelihood of receiving Tx2, though nonsignificant at *α *= 0.05. The proxy for poor performance status (HR = 0.82; *P* < 0.01) was significantly associated with lower likelihood of receiving Tx2. A longer time period between diagnosis and the beginning of Tx1 was associated with lower likelihood of proceeding to Tx2 (HR = 0.996; *P* < 0.01). Each additional 10-year increment of age at diagnosis (HR = 1.14; *P* < 0.01) and qualifying for State buy-in (HR = 1.21; *P* = 0.01) were associated with higher likelihood of receiving Tx2. Being married (HR = 0.87; *P* = 0.01) significantly decreased the likelihood of proceeding to Tx2. Hispanics (HR = 0.67; *P* < 0.01) were less likely to receive Tx2, as compared to non-Hispanic whites.

Comparing the impacts of patient-level factors on Tx1 versus Tx2, being older reduced the likelihood of receiving Tx1, yet increased the likelihood of receiving Tx2; Being married was associated with a greater likelihood of receiving Tx1 but a lower likelihood of receiving Tx2; State buy-in status was similarly positively associated with receiving Tx1 and negatively associated with receiving Tx2.

**Figure 3:**
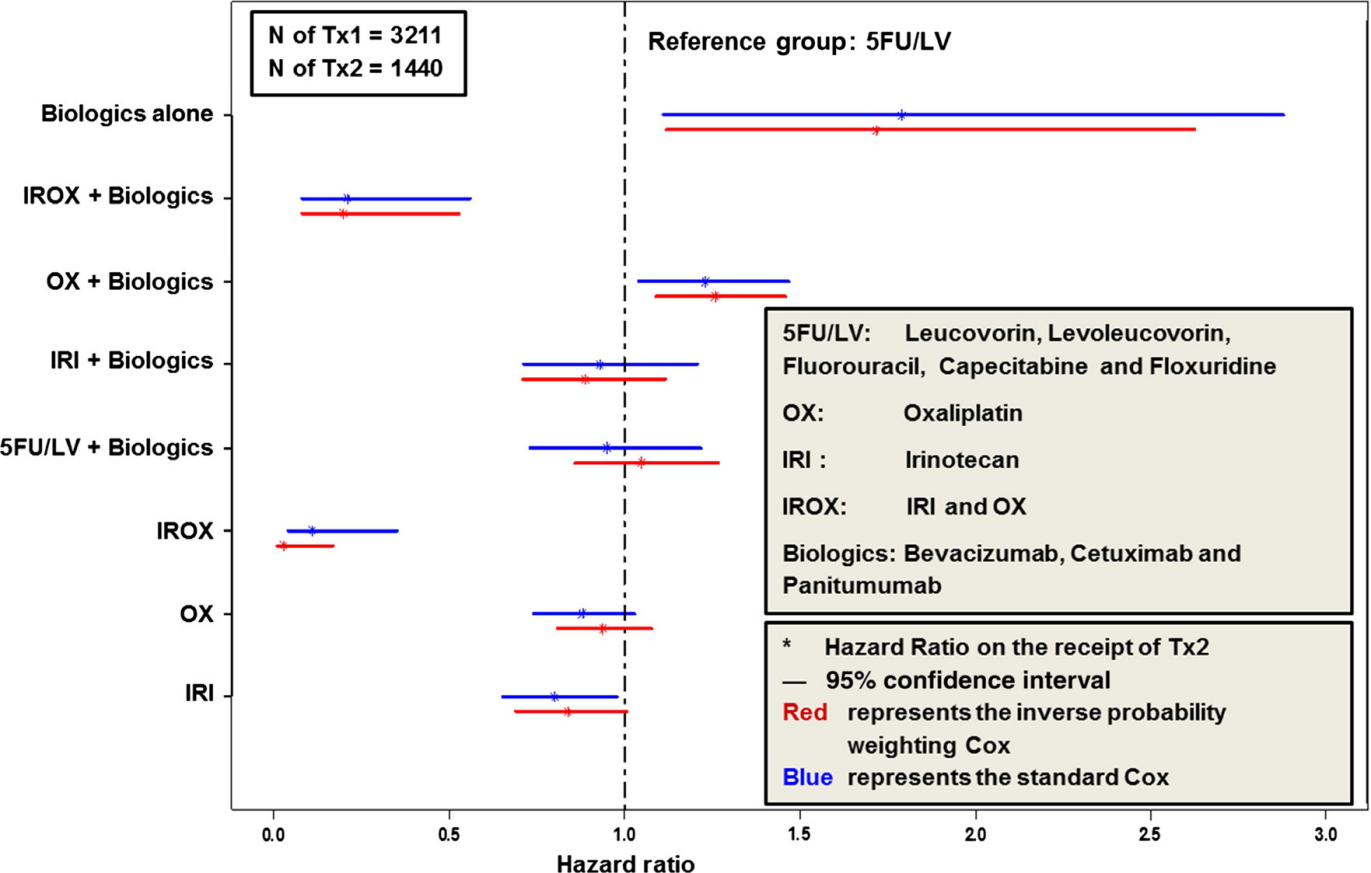
Impact of first-line treatment (Tx1) (other chemotherapy drugs/biologics vs. 5FU/LV) on the receipt of second-line treatment (Tx2).

Figure 3 compares the impacts of the specific regimen(s) used in Tx1 on the likelihood of proceeding to Tx2 for the standard Cox regression and IPW Cox regression. All other regimens were compared to 5FU/LV. Blue lines represent the 95% confidence intervals obtained by standard Cox regression and red lines represent the 95% confidence intervals obtained by IPW Cox regression. The impact of other regimens (compared to 5Fu/LV) on the receipt of Tx2 were robust when IPW was used in the Cox regression.

**Figure 4:**
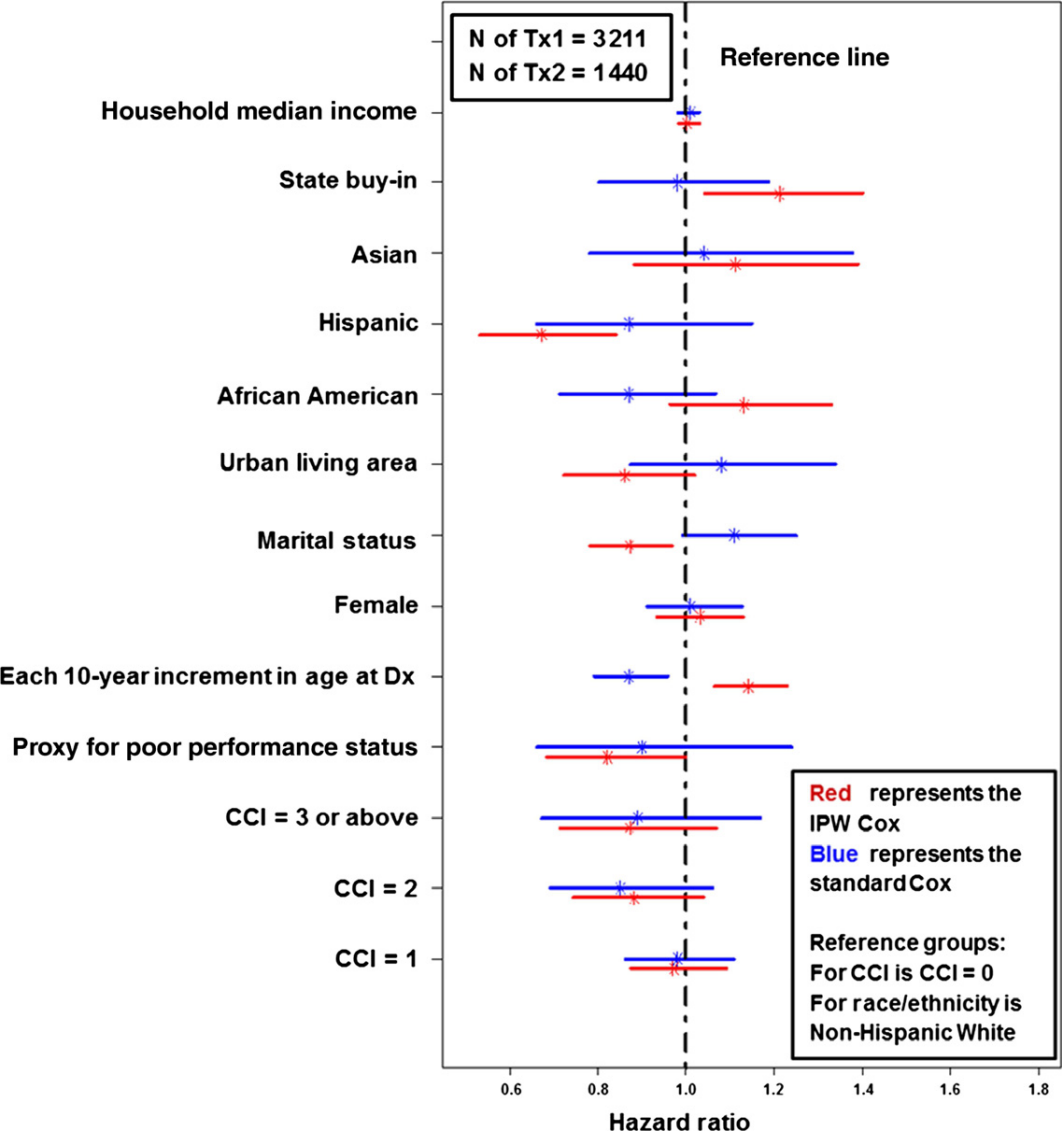
Impact of patient-level factors on the receipt of second-line treatment (Tx2).

Figure 4 compares the impacts of other patient-level factors on the receipt Tx2 between the standard Cox regression and the IPW Cox regression. There were significant changes between the two (from standard Cox to IPW Cox): (1) the proxy for poor performance status changed from nonsignificant to significant; (2) each 10-year increment in age changed from reducing to increasing the likelihood of receiving Tx2; (3) Being married changed from nonsignificant to a significantly lower likelihood of proceeding to Tx2; (4) Hispanic ethnicity changed from being nonsignificant to having a significant association with a lower likelihood of receipt of Tx2; (5) State buy-in changed from nonsignificant to significantly higher likelihood of proceeding to Tx2.

## Discussion

Guglielmi and Sobrero reviewed and summarized the available evidence from those RCTs that assessed Tx2 after the failure of Tx1 for advanced colorectal cancer 29. They concluded that: (1) Tx2 is superior to best supportive care alone; (2) following 5-FU failure, active regimens include IRI, OX, and IROX, with IROX appearing to be superior to IRI; (3) following IRI based first-line treatment, OX is in general the best choice, and the combination of OX plus BEV appears to be superior to OX alone; (4) following first-line OX, IRI is currently the most appropriate options. IRI plus CETUX should emerge as an effective regimen. However, factors that are associated with the receipt of Tx2 in clinical practice remain unclear.

Our analysis shows that the receipt of Tx2 depends on both the specific regimens in Tx1 and patient-level factors. Patients who receive IROX with/out a biologic for Tx1 are less likely to receive Tx2 as compared to those who start with 5FU/LV. This may be due to longer progression free survival associated with IROX. However, during the time frame of this study, there were limited options so patients who received IROX initially did not have other chemotherapy agents to try as Tx2 as they might in 2013. When patients receive IRI with/out biologics or OX alone as Tx1, they are no more or less likely to receive Tx2 as compared to those who start with 5FU/LV. However, in contrast to IROX, OX + biologics users are more likely to receive Tx2 as compared to those who receive 5FU/LV as Tx1.

In addition to Tx1, ethnicity and indicator for poor performance status also influence the receipt of Tx2. In particular, our results document that Hispanics and patients with an indicator of poor performance status are less likely to receive Tx2. This study also found that higher age is associated with lower likelihood of receiving Tx1 but higher likelihood of receiving Tx2, conditional on having received Tx1. Older patients are more likely to receive 5FU/LV in Tx1, which is associated with shorter Tx1 duration (Fig. 1).

Politano et al. reported that the only significant variable associated with receiving additional chemotherapy was failure to respond to the initial treatment 10. However, that study was a small retrospective analysis of second-line chemotherapy use, where patients were limited to those who received BEV as part of the initial treatment. As compared to their study where patients were recruited in a single medical center, our observational study discovered various clinical practice patterns regarding the use of regimens in Tx2 among elderly mCC patients. The management strategies of Tx2 in our sample can be quite different than the clinical trials. Therefore, this study is more likely to reflect the real world setting and can provide complementary information to policy makers as well as clinicians. Also, this study sample consists of elderly mCC patients with multiple comorbid conditions and includes patients from a broader spectrum of socioeconomic status. It is worth noting that 74% of the 77 patients in the Politano study eventually received Tx2, which is much higher than the 45% in this study.

One limitation of this study is that our results may not be generalizable to the nonelderly population. The identification of Tx2 is based on a claims-based algorithm that was developed and published by a team of clinicians, health services researchers, and programmers. The algorithm does not reflect off-label use or non-NCCN recommended drugs. Another potential group of patients who did not proceed to Tx2 and not captured by our analysis may include patients who (1) refused further treatment, (2) deemed not good candidates for treatment (have contraindications), (3) are untreated (should be treated, but do not receive chemotherapy). Lastly, we recognize that the IPW Cox regression may not entirely eliminate the correlation between receipt of Tx2 and survival probability.

## Conclusion

Among elderly mCC patients, the receipt of second-line treatment depends on the chemotherapy drugs/biologics received in the initial treatment. Patient with an indicator of poor performance status are less likely to receive second-line treatment. Hispanic ethnicity, but not African American or Asian race, is associated with lower use of second-line treatment. Advanced age is associated with lower rates of initial treatment receipt; however, conditional upon receiving first-line treatment, advanced age is associated with higher use of second-line treatment.
